# Supplementary material for: Intracranial EEG signals disentangle multi-areal neural dynamics of vicarious pain perception
Source: Nat Commun. 2024 Jun 18;15:5203. doi: 10.1038/s41467-024-49541-1 (PMC11189531; doi:10.1038/s41467-024-49541-1)
Supplement: Supplementary file 1 — Supplementary Information [file 41467_2024_49541_MOESM1_ESM.pdf]

Supplementary Information

Intracranial EEG signals disentangle multi-areal neural dynamics of vicarious pain perception

Huixin Tan[1,2,3], Xiaoyu Zeng[1,2,3], Jun Ni[1,2,3], Kun Liang[4], Cuiping Xu[5], Yanyang Zhang[6], Jiaxin Wang[1,2,3], Zizhou Li[1,2,3], Jiaxin Yang[1,2,3], Chunlei Han[4], Yuan Gao[4], Xinguang Yu[6], Shihui Han[7], Fangang Meng[4,8*], Yina Ma[1,2,3,8*]

[1] State Key Laboratory of Cognitive Neuroscience and Learning Beijing Normal University, Beijing, China

[2] IDG/McGovern Institute for Brain Research, Beijing Normal University, Beijing, China

[3] Beijing Key Laboratory of Brain Imaging and Connectomics, Beijing Normal University, Beijing, China

[4] Beijing Tiantan Hospital, Capital Medical University, Beijing, China

[5] Department of Functional Neurosurgery, Xuanwu Hospital, Capital Medical University, Beijing, China

[6] Department of Neurosurgery, Chinese PLA General Hospital, Beijing, China

[7] School of Psychological and Cognitive Sciences, PKU-IDG/McGovern Institute for Brain Research, Peking University, Beijing, China

[8] Chinese Institute for Brain Research, Beijing, China

* Correspondence should be addressed to:
Yina Ma, Ph.D. (Email: yma@bnu.edu.cn).

Supplementary Figures

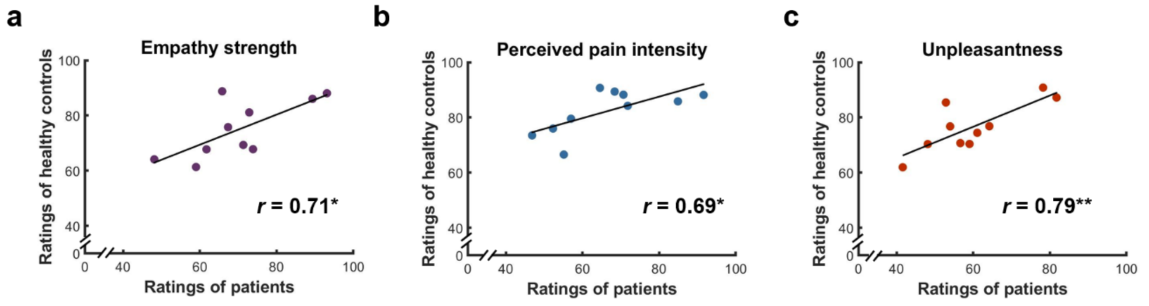

Supplementary Fig. 1 | *Similarity between patients and healthy controls in empathy-related ratings at the group level.* Patients and healthy controls exhibited similar response patterns, as evidenced by the high similarities in subjective ratings of empathic strength (**a**, $r = 0.71$, $p = 0.022$), perceived pain intensity (**b**, $r = 0.69$, $p = 0.027$), and unpleasantness (**c**, $r = 0.79$, $p = 0.007$) across painful stimuli ($n = 10$ trials). All results survived the FDR-correction across dimensions. Each circle represents a painful stimulus and the solid line represents the fitted regression line. $*p < 0.05$, $**p < 0.01$. Source data are provided as a Source Data file.

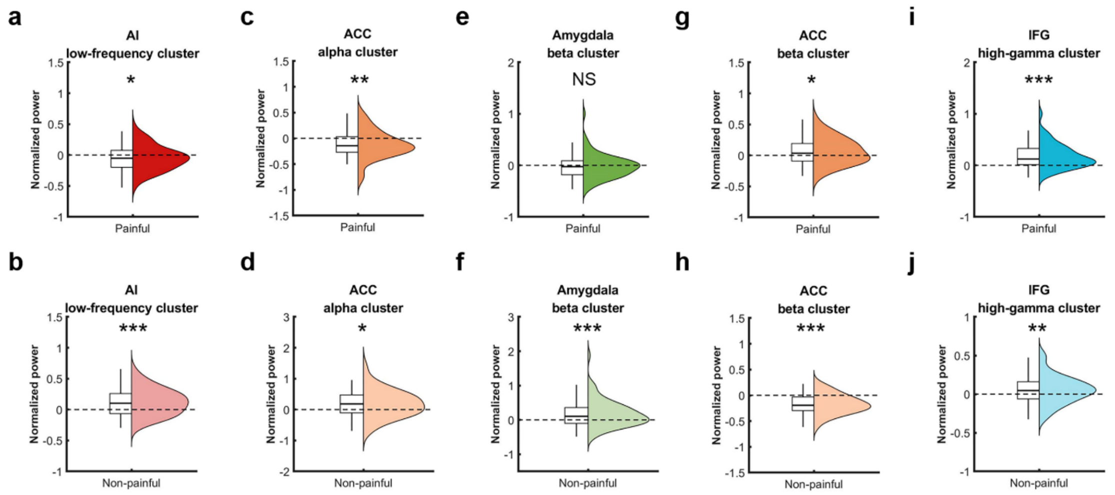

Supplementary Fig. 2 | ***Spectro-temporal power for each condition in the AI, ACC, amygdala and IFG***. Split-half violin plots indicate the probability density of the averaged power across time-frequency points that exhibited significant conditional differences (significant clusters in Fig. 2e-h) in each condition for the AI low-frequency cluster (*n* = 98 channels, **a,** t_{97} = -2.44, *p* = 0.016, **b**, t_{97} = 5.76, *p* = 9.94×10^{-8}), ACC alpha cluster (*n* = 40 channels, **c,** t_{39} = -2.94, *p* = 0.006, **d**, t_{39} = 2.69, *p* = 0.010), amygdala beta cluster (*n* = 68 channels, **e,** t_{67} = -0.63, *p* = 0.534, **f**, t_{67} = 3.76, *p* = 3.57×10^{-4}), ACC beta cluster (*n* = 40 channels, **g,** t_{39} = 2.02, *p* = 0.050, **h**, t_{39} = -5.70, *p* = 1.35×10^{-6}), and IFG high-gamma cluster (*n* = 91 channels, **i,** t_{90} = 7.09, *p* = 2.89×10^{-10}, **j**, t_{90} = 2.90, *p* = 0.005) (two-sided one-sample *t*-tests). The left boxplots showed the interquartile range of 50% with lower and upper quartile limits at 25% and 75%, respectively. Within the boxplot, the middle line represents the median and whiskers are extended to the most extreme data points that are no more than 1.50 times the interquartile range. *$p < 0.05$, **$p < 0.01$, ***$p < 0.001$, NS, not significant. Source data are provided as a Source Data file.

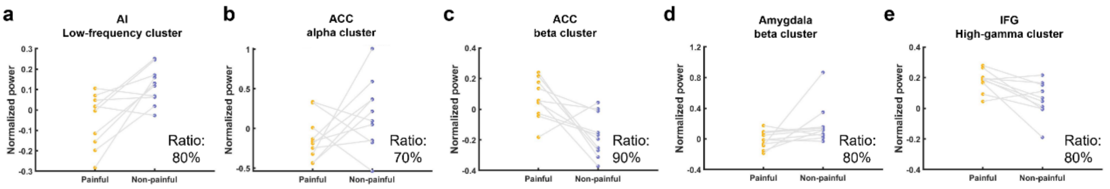

Supplementary Fig. 3 | ***Consistent spectro-temporal power patterns in the AI, ACC, amygdala, and IFG across trials.*** (**a-d**) We averaged power across channels and time-frequency points that exhibited significant conditional differences (significant clusters in Fig. 2e-h) for the AI **(a)**, ACC **(b, c)**, amygdala **(d)**, and IFG **(e).** The reported neural patterns of these clusters were consistently observed across the majority of trials (with the ratio of trials showing consistent patterns depicted at the bottom right corner of the figure). Each orange (painful) and violet (non-painful) dot indicate single-trial normalized power and grey lines show power differences between matched painful and non-painful stimuli. Source data are provided as a Source Data file.

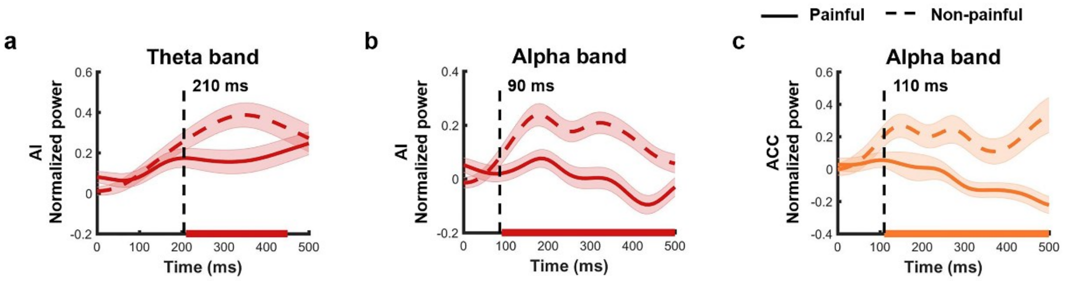

Supplementary Fig. 4 | *Temporal profiles of theta and alpha power in the AI and ACC.* The perception of painful (*vs.* non-painful) stimuli decreased theta power in the AI from 210 ms to 450 ms after stimulus onset (**a**, $p = 0.009$). Empathy-induced alpha power decreases were observed at 90 ms after stimulus onset in the AI (indexed by a significant decrease in the time window of 90 to 500 ms (**b**, $p < 0.001$), and at 110 ms after stimulus onset in the ACC (indicated by a significant decrease in the time window of 110 to 500 ms (**c**, $p < 0.001$). The theta or alpha power averaged across all channels within the AI ($n = 98$ channels) or ACC ($n = 40$ channels) was plotted as a function of time. Time points with significant conditional power differences are highlighted with horizontal lines on the *x*-axis (one-tailed paired-*t* test for each timepoint, corrected $p < 0.01$, 1000 permutations, using a cluster-based permutation test with at least maintenance for consecutive 50ms periods). Dashed vertical lines indicate when empathy-related power decreases occurred during painful compared to non-painful conditions. Solid (painful condition) and dashed (non-painful condition) lines [shadows] indicate the mean [standard error] power across all channels for each time point. Source data are provided as a Source Data file.

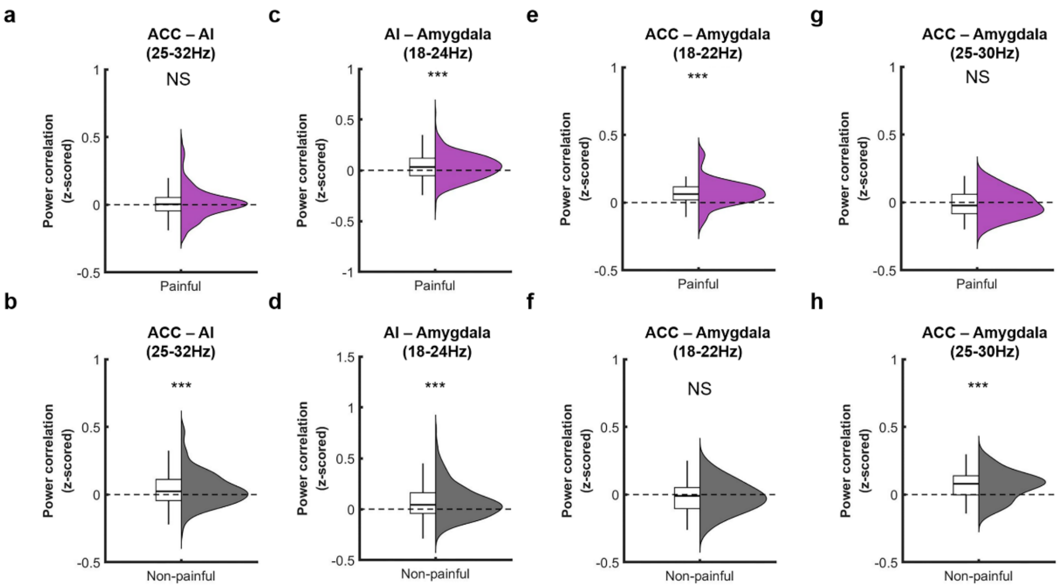

Supplementary Fig. 5 | ***Power correlations between ACC, AI and amygdala within each condition.*** Purple (grey) split-half violin plots indicate the probability density of the averaged Fisher-z-transformed power correlation values across frequency ranges that significantly differentiated between painful and non-painful stimuli (significant clusters in Fig. 3a-c) in the painful (non-painful) condition for ACC-AI **(**n = 234 channel pairs, **a,** $t_{233} = 1.73$, $p = 0.085$, **b**, $t_{233} = 5.05$, $p = 8.98\times10^{-7}$**)**, AI-amygdala **(**n = 300 channel pairs, **c,** $t_{299} = 4.63$, $p = 5.45\times10^{-6}$,**d,** $t_{299} = 7.56$, $p = 4.87\times10^{-13}$**)**, and ACC-amygdala **(**n = 72 channel pairs, **e,** $t_{71} = 6.42$, $p = 1.34\times10^{-8}$, **f**, $t_{71} = -1.16$, p = 0.248, **g**, $t_{71} = -0.82$, $p = 0.416$, **h**, $t_{71} = 5.50$, $p = 5.62\times10^{-7}$**)** (two-sided one-sample t-tests). The left boxplots showed the interquartile range of 50% with lower and upper quartile limits at 25% and 75%, respectively. Within the boxplot, the middle line represents the median and whiskers are extended to the most extreme data points that are no more than 1.50 times the interquartile range. ***$p < 0.001$, NS, not significant. $\beta_{low/high}$, low (high) frequency range within the beta band. Source data are provided as a Source Data file.

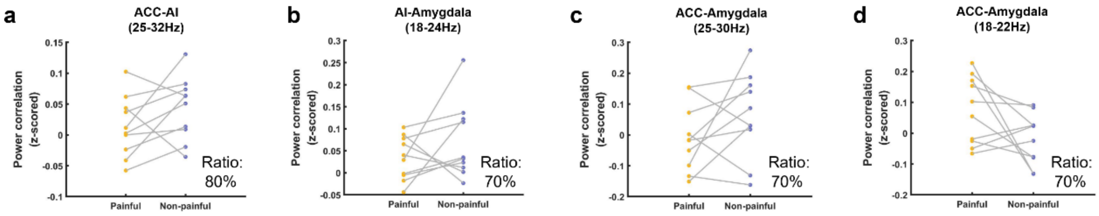

Supplementary Fig. 6 | ***Consistent power correlations patterns between ACC, AI, and amygdala across trials.*** (**a-d**) We averaged Fisher-z-transformed power correlation values across channel-pairs and frequency ranges that significantly differentiated between painful and non-painful stimuli (significant clusters in Fig. 3a-c) for ACC-AI **(a)**, AI-amygdala **(b)**, and ACC-amygdala **(c-d).** The reported neural patterns of these region pairs were consistently observed across the majority of trials (with the ratio of trials showing consistent patterns depicted at the bottom right corner of the figure). Each orange (painful) and violet (non-painful) dot indicate single-trial Fisher-z-transformed power correlation values and grey lines show power correlation differences between matched painful and non-painful stimulus. Source data are provided as a Source Data file.

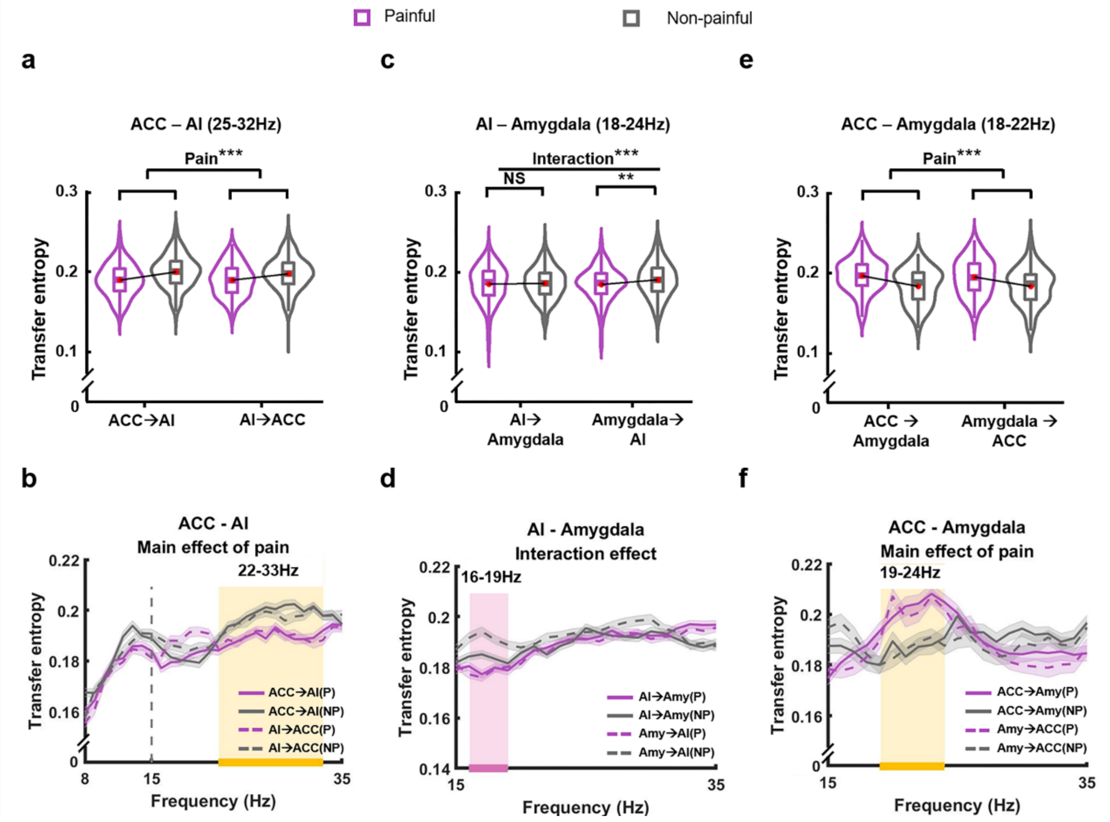

Supplementary Fig. 7 | ***Transfer entropy (TE) between ACC, AI and amygdala.*** We observed the main effect of pain on TE values for ACC-AI (*n* = 234 channel pairs, **a**, averaged TE values across 25-32 Hz, $F_{1, 233}$ = 30.04, p = 1.10 × 10^{-7}; **b**, a significant cluster of 22-33 Hz among 8-35 Hz, $p < 0.001$**),** the interaction effect on TE values for AI-Amygdala (*n* = 300 channel pairs, **c,** averaged TE values across 18-24 Hz, $F_{1, 299}$ = 13.67, p = 2.59 × 10^{-4}, effect of pain for AI-to-amygdala: t_{299} = -0.48, p = 0.629, effect of pain for amygdala-to-AI: t_{299} = -3.25, p = 0.001; **d**, a significant cluster of 16-19 Hz among 15-35 Hz, $p < 0.001$**)**, and the main effect of pain on TE values for ACC-Amygdala (*n* = 72 channel pairs, **e**, averaged TE values across 18-22 Hz, $F_{1, 71}$ = 20.14, p = 2.72 × 10^{-5}; **f**, a significant cluster of 19-24 Hz among 15-35 Hz, $p < 0.001$). For (**a**/**c**/**e**), the inner boxplots showed the interquartile range of 50% with lower and upper quartile limits at 25% and 75%, respectively. Within the boxplot, the middle line represents the median, red points represent the mean, and whiskers are extended to the most extreme data points that are no more than 1.50 times the interquartile range. Solid lines between violin plots start from the mean and reflect conditional TE differences for each direction. **$p < 0.01$, ***$p < 0.001$, NS, not significant. For (**b**/**d**/**f**), purple (painful condition) and gray (non-painful condition) lines (shadows) indicate the mean (standard error) of TE across all channel pairs. Solid and dashed lines represented different directions respectively. Significant main effect of pain (interaction effect) is highlighted with light-orange (pink) rectangles and orange (pink) horizontal lines on the axis (two-sided, cluster-based permutation tests, corrected p < 0.01). Dashed vertical lines indicate boundaries between frequency bands. P = painful, NP = non-painful, Amy = amygdala. Source data are provided as a Source Data file.

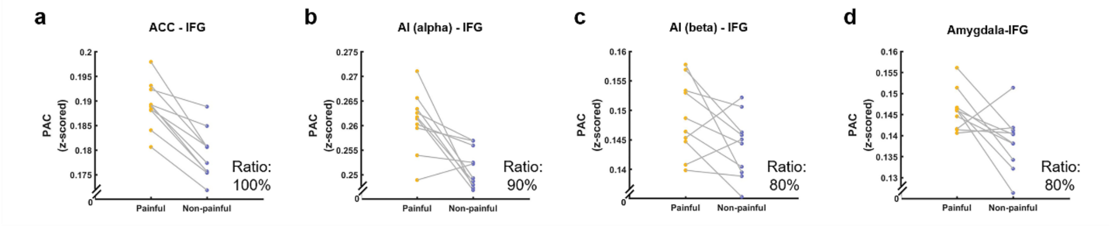

Supplementary Fig. 8 | ***Consistent phase-amplitude coupling (PAC) patterns ACC/AI/amygdala and IFG across trials.*** (**a-d**) We averaged Fisher-z-transformed PAC values across spectral pairs that significantly differentiated between painful and non-painful stimuli (significant clusters in Fig. 4a-c) for ACC-IFG **(a)**, AI-IFG **(b-c)**, and amygdala-IFG **(d)**. The reported neural patterns of these region pairs were consistently observed across the majority of trials (with the ratio of trials showing consistent patterns depicted at the bottom right corner of the figure). Each orange (painful) and violet (non-painful) dot indicate single-trial Fisher-z-transformed PAC values and grey lines show PAC differences between matched painful and non-painful stimuli. Source data are provided as a Source Data file.

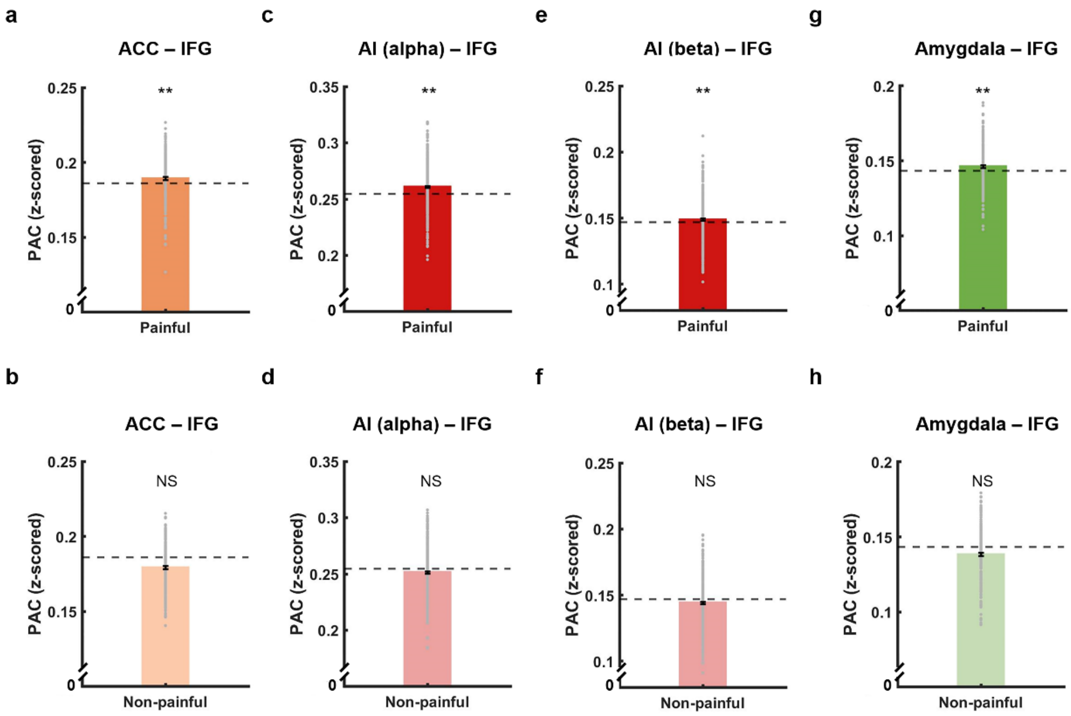

Supplementary Fig. 9 | *Phase-amplitude coupling (PAC) between ACC/AI/amygdala and IFG*. Bar plots indicate the averaged Fisher-z-transformed PAC values across spectral pairs that significantly differentiated between painful and non-painful stimuli (significant clusters in Fig. 4a-c) in each condition for ACC-IFG **(a, b**, n = 219 channel pairs**)**, AI-IFG **(c-f**, n = 459 channel pairs**)**, and amygdala-IFG **(g, h**, n = 254 channel pairs**)**. We assessed the significance of PAC values for each condition using permutation tests. Within each permutation, we divided the amplitude time series at a random time-point into two parts and then reversed their order to generate permutated amplitude time series[1]. The same analyses of PAC were then applied to the permutated data, and the resulting PAC values were averaged across all channel-pairs to generate a distribution of permuted PAC values (200 permutations). The PAC value of either the painful or non-painful condition was compared to this permutation distribution, and it was considered significant if it exceeded 95% of this permutation distribution (one-sided)[2,3]. Data are presented as mean values ± SE. Dots represent individual channel pairs. Horizontal dashed lines correspond to a threshold for a p value of 0.05. **$p < 0.01$, NS, not significant. Source data are provided as a Source Data file.

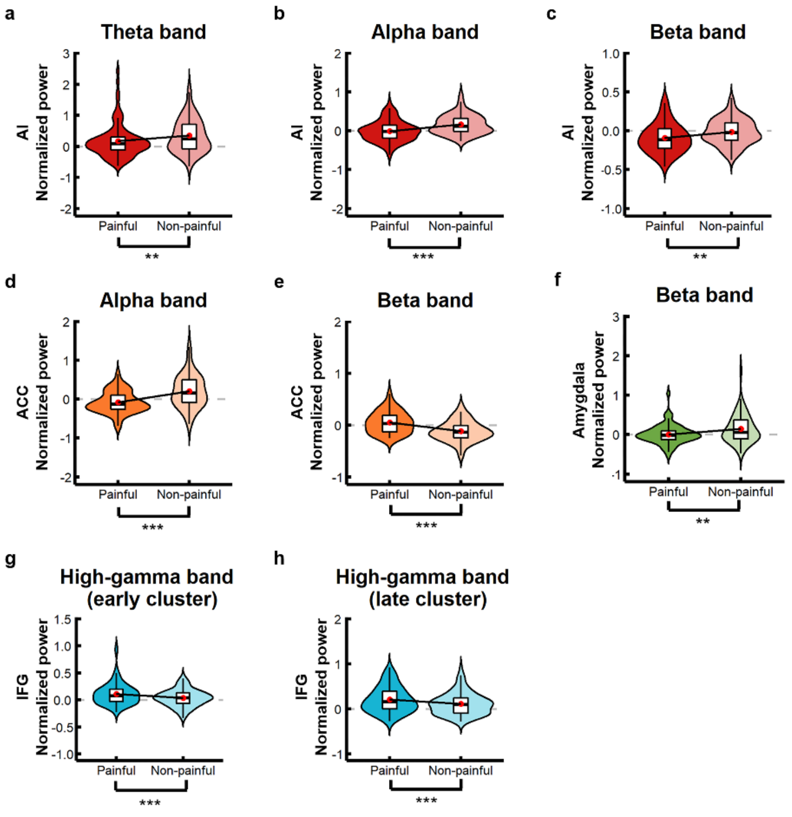

Supplementary Fig. 10 | *Empathy-related spectro-temporal power based on the analysis of linear mixed-effect models.* We averaged the power in the time window that significantly differed between painful and non-painful stimuli for each significant frequency band (i.e., significant clusters in Fig. 2i-l and Supplementary Fig. 4). Similar to previous studies[4,5], power were entered into linear mixed-effect models (two-tailed) that included the fixed-effect of pain and random effects of patients and channel nested within patients. Empathy-related spectro-temporal power changes remained reliable in the AI (n(trial)/n(channel) = 1904/98, **a,** theta band: β = -0.17 ± 0.05, t_{1902} = -3.19, p = 0.001; **b**, alpha band: β = -0.16 ± 0.04, t_{1902} = -4.49, p = 7.50×10^{-6}; **c**, beta band: β = -0.08 ± 0.03, t_{1902} = -2.77, p = 0.006), ACC (n(trial)/n(channel) = 785/40, **d**, alpha band: β = -0.28 ± 0.06, t_{783} = -4.44, p = 1.05×10^{-5}; **e**, beta band: β = 0.17 ± 0.05, t_{783} = 3.70, p = 2.28×10^{-4}), amygdala (n(trial)/n(channel) = 1327 /68, **f,** beta band: β = -0.13 ± 0.05, t_{1325} = -2.82, p = 0.005), and IFG (n(trial)/n(channel) = 1787/91, **g**, high-gamma band, early cluster (60-340 ms): β = 0.07 ± 0.02, t_{1785} = 3.89, p = 1.05×10^{-4}; **h**, high-gamma band, late cluster (380-470 ms): β = 0.10 ± 0.02, t_{1785} = 3.88, p = 1.07×10^{-4}). Violin plots indicate the probability distribution of power with inner boxplots showing the interquartile range of 50% (lower and upper quartile limits are 25% and 75%, respectively). In the boxplot, the middle line represents the median, the red point represents the mean, and whiskers are extended to the most extreme data points that are no more than 1.50 times the interquartile range. Solid lines between violin plots start from the mean and reflect conditional differences in the mean value of power. **$p < 0.01$, ***$p < 0.001$. Source data are provided as a Source Data file.

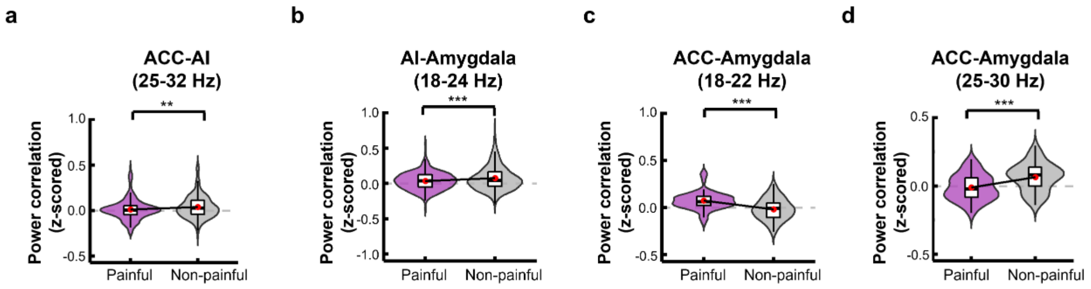

Supplementary Fig. 11 | ***Low-frequency synchronization between the ACC, AI, and amygdala based on the analysis of linear mixed-effect models.*** We averaged the Fisher-z-transformed power-correlation (PC) values of frequency ranges that significantly differentiated between painful and non-painful stimuli (i.e., significant clusters in Fig. 3a-c). We conducted linear mixed-effect models (two-tailed) that included the fixed-effect of pain and random effects of patients and channel pairs nested within patients to compare PC values between painful and non-painful conditions. **(a-d)** We validated decreased beta synchronization between ACC and AI (*n*(trial)/*n*(channel pair) = 4475/234, **a**, 25-32 Hz: β = -0.03 ± 0.01, t_{4473} = -3.18, p = 1.47 × 10^{-3}) and between AI and amygdala (*n*(trial)/*n*(channel pair) = 5703/300, **b**, 18-24Hz: β = -0.04 ± 0.01, t_{5701} = -4.32, p = 1.60 × 10^{-5}), increased ACC-amygdala synchronization at a lower frequency range within the beta band (*n*(trial)/*n*(channel pair) = 1382/72, **c**, 18-22 Hz: β = 0.09 ± 0.02, t_{1360} = 4.63, p = 4.00 × 10^{-6}) but decreased ACC-amygdala synchronization at the upper frequency range within the beta band (**d**, 25-30 Hz: β = -0.08 ± 0.02, t_{1360} = -4.18, p = 3.16 × 10^{-5}). Violin plots indicate the probability distribution of PC (z-scored) with inner boxplots showing the interquartile range of 50% (lower and upper quartile limits are 25% and 75%, respectively). In the boxplot, the middle line represents the median, the red point represents the mean, and whiskers are extended to the most extreme data points that are no more than 1.50 times the interquartile range. Solid lines between violin plots start from the mean and reflect conditional differences in the mean value of power correlations. **p < 0.01, ***p < 0.001. Source data are provided as a Source Data file.

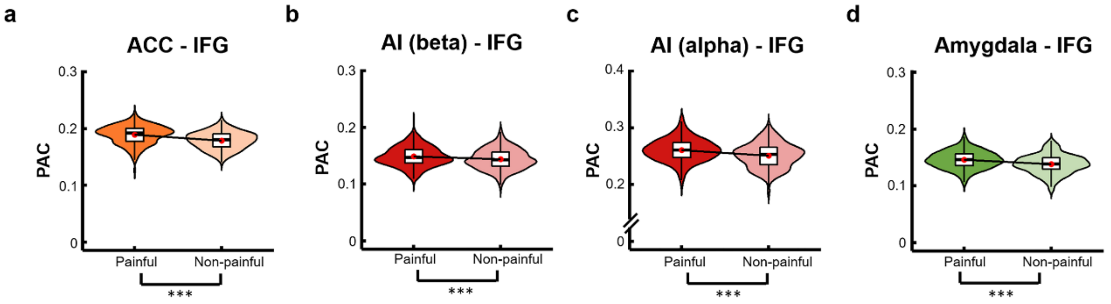

Supplementary Fig. 12 | *ACC/AI/amygdala-IFG phase amplitude coupling based on the analysis of linear mixed-effect models.* We averaged the Fisher-z-transformed phase-amplitude coupling (PAC) values of the frequency ranges that significantly differentiated between painful and non-painful stimuli (i.e., significant clusters in Fig. 4a-c). PAC values were entered into linear mixed-effect models that included the fixed-effect of pain and random effects of patients and channel pairs nested within patients (two-tailed). The high-gamma amplitude of the IFG remained phase-locked to the beta phase of the ACC (*n*(trial)/*n*(channel pair) = 4229/219, **a**, $\beta = 0.010 \pm 0.001$, $t_{4227} = 7.89$, $p = 3.77 \times 10^{-15}$), the beta phase of AI (*n*(trial)/*n*(channel pair) = 8892/459, **b**, $\beta = 0.005 \pm 0.001$, $t_{8890} = 4.15$, $p = 3.30 \times 10^{-5}$), the alpha phase of AI (*n*(trial)/*n*(channel pair) = 8892/459, **c**, $\beta = 0.009 \pm 0.001$, $t_{8890} = 7.58$, $p = 3.77 \times 10^{-14}$), and the beta phase of amygdala (*n*(trial)/*n*(channel pair) = 4856/254, **d**, $\beta = 0.008 \pm 0.001$, $t_{4854} = 6.28$, $p = 3.79 \times 10^{-10}$). Violin plots indicate the probability distribution of Fisher-z-transformed PAC values with inner boxplots showing the interquartile range of 50% (lower and upper quartile limits are 25% and 75%, respectively). In the boxplot, the middle line represents the median, the red point represents the mean and whiskers are extended to the most extreme data points that are no more than 1.50 times the interquartile range. Solid lines between violin plots start from the mean and reflect conditional differences in the mean value of phase-amplitude coupling. ***$p < 0.001$. Source data are provided as a Source Data file.

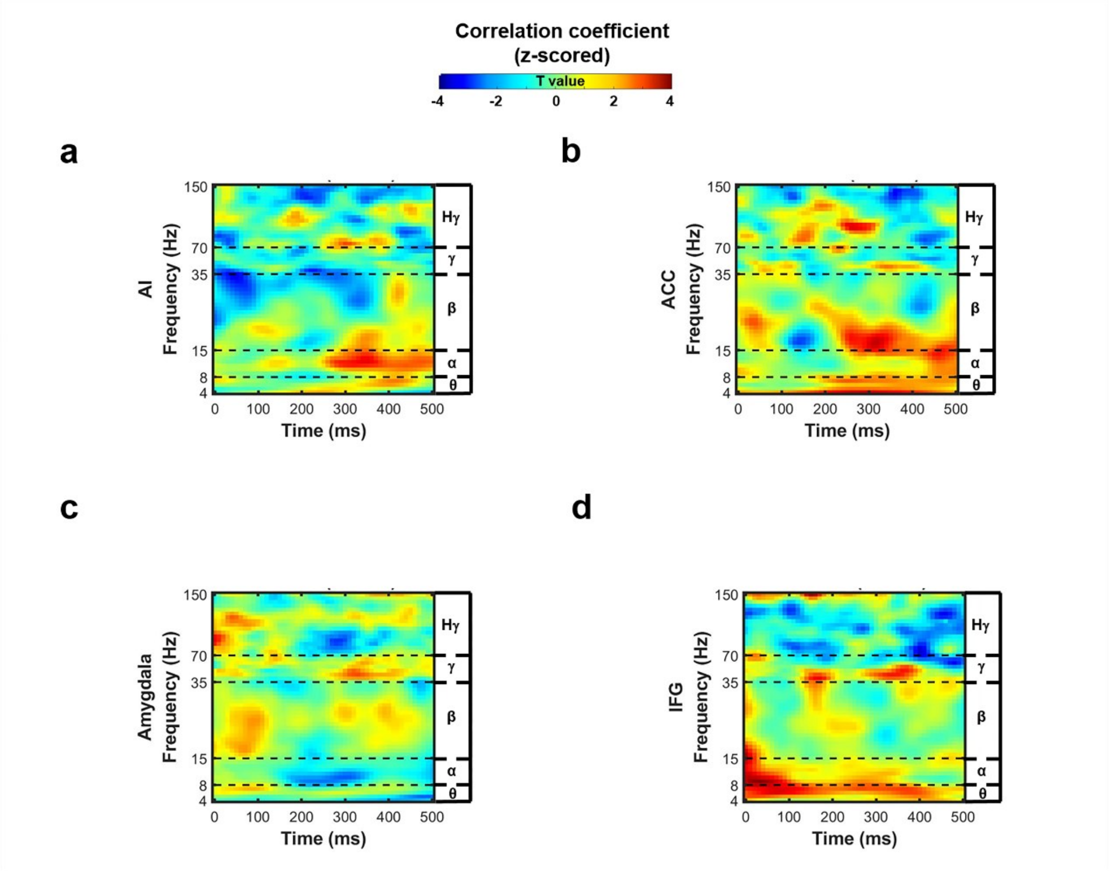

Supplementary Fig. 13 | *The correlation between power and the arousal level.* Ratings of arousal induced by the experimental stimuli showed excellent between-rater reliabilities (ICC = 0.95), enabling further analysis to correlate patients' arousal rating scores and neural responses. For each channel and time-frequency point, we correlated the power and arousal differences between matched painful and non-painful pairs of stimuli. No significant results were found in the AI (**a**, n = 70 channels), ACC (**b**, n = 36 channels), amygdala (**c**, n = 46 channels), and IFG (**d**, n = 71 channels) (two-sided one-sample t-tests of the Fisher-z-transformed correlation coefficients for each time-frequency point, 1000 permutations, using cluster-based permutation test to correct for multiple comparisons, all clusters corrected ps > 0.01). As no significant clusters were detected, we decided to conclude this analysis at this stage without performing additional linear mixed-effect model analyses. Warmer colors indicate higher t values. Horizontal dashed lines indicate boundaries between frequency bands and Hγ represents the high-gamma band. Source data are provided as a Source Data file.

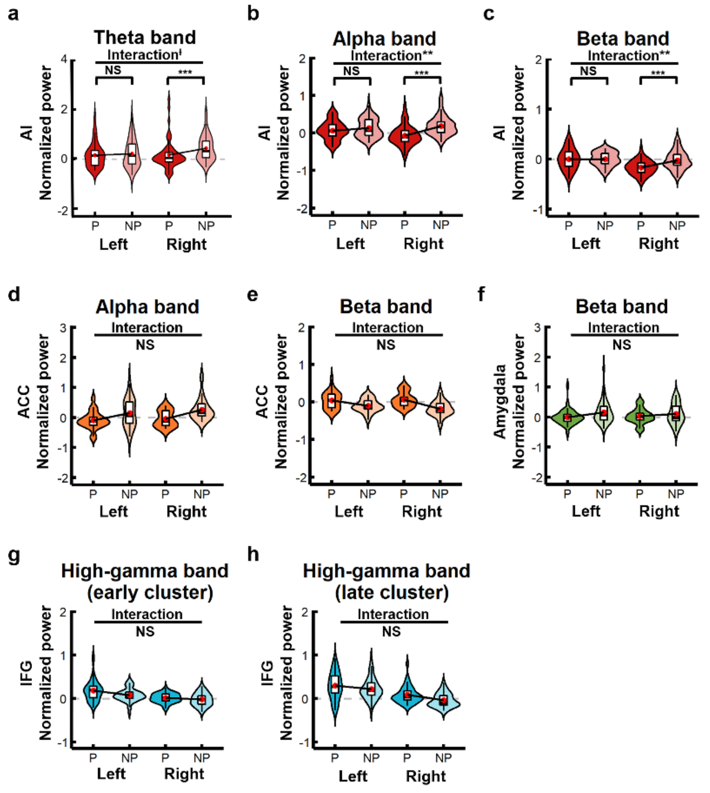

Supplementary Fig. 14 | *Hemispheric differences in empathy-related spectro-temporal power.* We categorized channels into left-hemisphere ($x < 0$) and right-hemisphere channels ($x > 0$) (channels whose x = 0 were excluded; Left/Right: **a-c,** n = 44/54 channels; **d-e,** n = 22/17 channels; **f,** n = 43/25 channels; **g-h,** n = 53/38 channels). We then averaged power in the time window that significantly differed between painful (P) and non-painful (NP) stimuli (i.e., significant clusters in Fig. 2i-l and Supplementary Fig. 4). Power values were entered into two-tailed mixed-effect ANOVAs, followed by planned two-tailed paired *t-tests* to compare painful and non-painful conditions separately on left-hemisphere and right-hemisphere channels. We observed a hemispheric difference in the AI low-frequency power, reflected by significant or marginally significant Pain × Hemisphere interaction effects (**a,** $F_{1,96}$ = 3.70, p = 0.057; **b**, $F_{1,96}$ = 9.93, p = 0.002; **c**, $F_{1,96}$ = 9.32, p = 0.003) as the decreased low-frequency power in the AI was only observed in the right hemisphere (**a,** t_{53} = -3.88, $p = 2.95 \times 10^{-4}$; **b**, t_{53} = -6.59, $p = 2.10 \times 10^{-8}$; **c**, t_{53} = -4.63, $p = 2.37 \times 10^{-5}$) but not left hemisphere (**a,** t_{43} = -0.88, p = 0.382; **b**, t_{43} = -1.34, p = 0.188; **c**, t_{43} = 0.01, p = 0.989). We did not observe significant hemispheric differences in other regions (**d**, $F_{1,37}$ = 0.22, p = 0.641; **e**, $F_{1,37}$ = 1.35, p = 0.252; **f**, $F_{1,66}$ = 0.96, p = 0.330; **g**, $F_{1,89}$ = 2.37, p = 0.127; **h**, $F_{1,89}$ = 1.33, p = 0.252). Violin plots indicate the probability distribution of power with inner boxplots showing the median and first and third quartiles. In the boxplot, the red point represents the mean, and whiskers are extended to the most extreme data points that are no more than 1.50 times the interquartile range. Solid lines between violin plots reflect conditional differences in the mean power. **$p < 0.01$, ***$p < 0.001$, ɫ, marginally significant, NS, not significant. Source data are provided as a Source Data file.

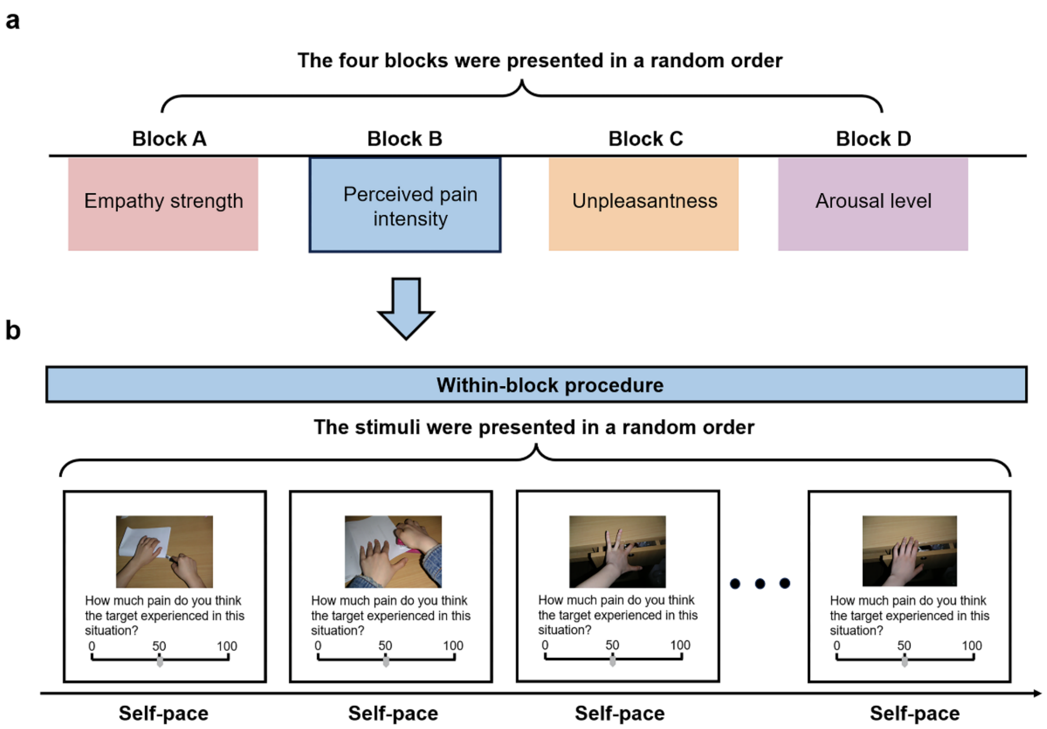

Supplementary Fig. 15 | *The procedure of the post-iEEG session*. a) We measured ratings for three dimensions related to empathy on separate blocks: i) the strength of empathic responses (**Block A**; with the question "How strongly do you feel empathic towards the target's pain?"; 0 = not at all, 100 = extremely strong); ii) the intensity of perceived pain in others (**Block B**; with the question "How much pain do you think the target experienced in this situation?"; 0 = not at all, 100 = extremely painful); iii) their own unpleasantness (**Block C**; with the question "How unpleasant do you feel when viewing the stimulus"; 0 = not at all, 100 = extremely unpleasant). To control for arousal levels, we also asked participants to report their arousal levels for each stimulus (**Block D**; with the question "How intense is your emotional response induced by this picture?"; 0 = extremely calm, 100 = extremely strong). The four blocks were presented in a random order. **b)** Within each block, we presented all stimuli in a random order and participants reported their ratings of each stimulus at their own pace (illustrated with an example of rating perceived pain intensity, **Block B**).

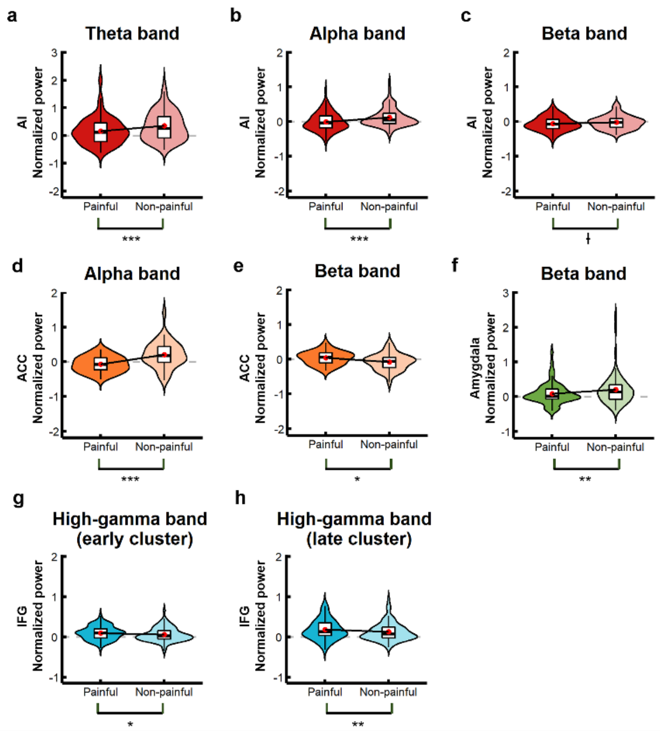

Supplementary Fig. 16 | ***Empathy-related spectro-temporal power when using the closest-white-matter referencing scheme.*** The iEEG signals were referenced to their nearest white-matter neighbors. We then averaged power in the time window that significantly differed between painful and non-painful stimuli (i.e., significant clusters in Fig. 2i-l and Supplementary Fig. 4) and compared power between painful and non-painful conditions in order to further confirm the observed power changes. Given that the directionality of the conditional differences has already been shown in the previous analyses based on the white-matter-average referencing scheme (Fig. 2i-l and Supplementary Fig. 4 and 10), one-sided paired *t*-tests were adopted for the following analyses (**a-c**, $n = 98$ channels; **d-e**, $n = 40$ channels; **f**, $n = 68$ channels; **g-h**, $n = 91$ channels). The empathy-related spectro-temporal power alternations replicated the main findings reported in the main text (**a,** theta band of AI: $t_{97} = -3.61$, $p = 2.46\times10^{-4}$; **b,** alpha band of AI: $t_{97} = -3.63$, $p = 2.26\times10^{-4}$,; **d,** alpha band of ACC: $t_{39} = -4.57$, $p = 2.41\times10^{-5}$; **e,** beta band of ACC: $t_{39} = 2.24$, $p = 0.016$; **f,** beta band of amygdala: $t_{67} = -2.91$, $p = 0.002$; **g,** high-gamma band of IFG, early cluster (60-340 ms): $t_{90} = 2.14$, $p = 0.018$; **h,** high-gamma band of IFG, late cluster (380-470 ms): $t_{90} = 2.94$, $p = 0.002$). The conditional power difference of the beta band in AI became marginally significant (**c**, beta band of AI: $t_{97} = -1.46$, $p = 0.073$). Violin plots indicate the probability distribution of power with inner boxplots showing the interquartile range of 50% (lower and upper quartile limits are 25% and 75%, respectively). In the boxplot, the middle line represents the median, the red point represents the mean, and whiskers are extended to the most extreme data points that are no more than 1.50 times the interquartile range. Solid lines between violin plots reflect conditional differences in the mean value of power. $*p < 0.05$, $**p < 0.01$, $***p < 0.001$, ł, marginally significant. Source data are provided as a Source Data file.

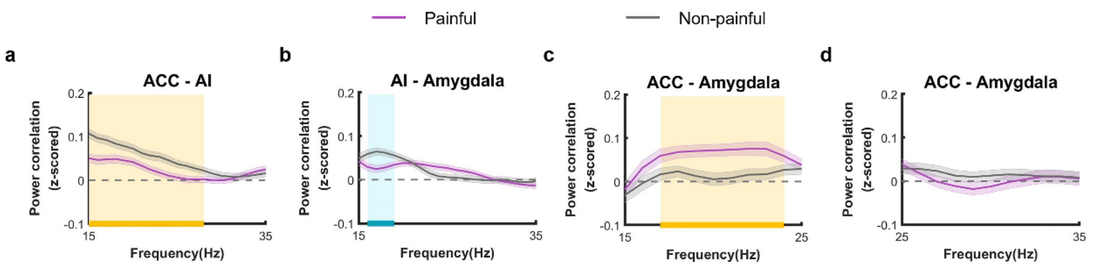

Supplementary Fig. 17 | *The low-frequency synchronization between ACC, AI, and amygdala using the closest-white-matter referencing scheme.* The iEEG signals were referenced to their nearest white-matter neighbors. We compared power correlation (PC) values between painful and non-painful conditions in the frequency bands that showed significant PC differences between painful and non-painful conditions using the white-matter-average referencing scheme (see Fig. 3a-c) for ACC-AI (**a**, n = 234 channel pairs), AI-amygdala (**b**, n = 300 channel pairs) and ACC-amygdala (**c**, n = 72 channel pairs) to further confirm our findings of power correlations. Since the directionality of the conditional differences has already been shown in the previous analyses (Fig. 3a-c and Supplementary Fig. 11), one-sided paired t-tests were adopted for the following analyses (paired-t test for each frequency, using cluster-based permutation test to correct for multiple comparisons). Notably, as previous analyses have revealed distinct ACC-amygdala PC patterns in the low and high frequency ranges within the beta band (β_{low} and β_{high}) (Fig. 3a-c), here we conducted separate PC analyses for low-beta band (15-25 Hz) and high-beta band (25-35 Hz)[6,7] between ACC and amygdala. Similarly, we found that perception of painful stimuli elicited weaker beta synchronization between ACC and AI (**a**, $p <$ 0.001) but stronger low-beta coupling between ACC and amygdala (**c**, p = 0.002). Applying a less conservative cluster threshold of corrected $p < 0.05$, similar response patterns were replicated for the beta synchronization between AI and amygdala (**b**, p = 0.021). Although the high-beta coupling strength between the ACC and amygdala became comparable for painful and non-painful conditions (all clusters corrected $ps >$ 0.05), we observed a similar trend with weaker high-beta coupling in the painful condition (**d**). Significant clusters with the threshold of corrected $p < 0.01$ ($p < 0.05$) are highlighted with light-orange (light-blue) rectangles and orange (blue) horizontal lines on the axis. Purple (painful condition) and grey (non-painful condition) lines (shadows) indicate the mean (standard error) frequency-resolved PC across all channel pairs. Source data are provided as a Source Data file.

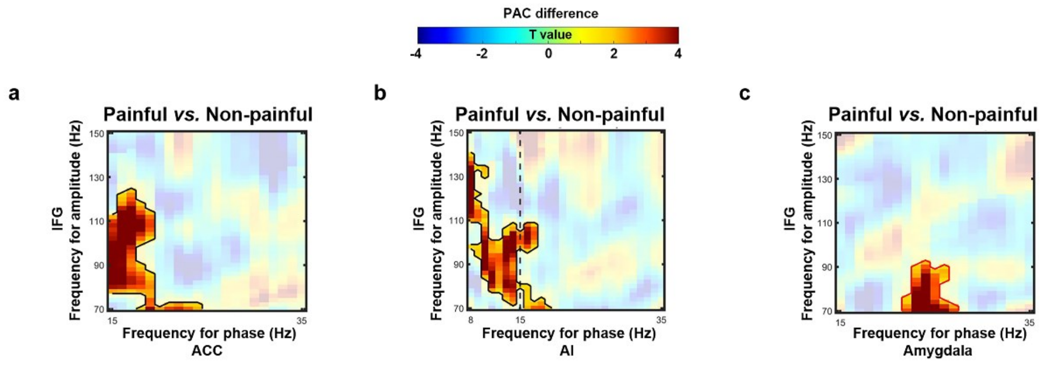

Supplementary Fig. 18 | ***ACC/AI/Amygdala-IFG phase-amplitude coupling when using the closest-white-matter referencing scheme.*** The iEEG signals were referenced to their nearest white-matter neighbors. We compared the ACC/AI/amygdala-IFG phase-amplitude coupling (PAC) values between the painful and non-painful conditions within the frequency bands that showed significantly increased PAC in the white-matter-average re-referencing scheme (Fig. 4). Since the directionality of the conditional differences has already been shown in the previous analyses (Fig. 4 and Supplementary Fig. 12), one-sided paired *t*-tests were adopted for the following analyses (paired-*t* test for each frequency pair, using cluster-based permutation test to correct for multiple comparisons). We found significant clusters with stronger coupling between the high-gamma amplitude of the IFG and the beta phase of the ACC (**a**, $n = 219$ channel pairs, $p < 0.001$) and the alpha (slightly extended to beta) phase of AI (**b**, $n = 459$ channel pairs, $p = 0.001$). Applying a less conservative cluster threshold of corrected $p < 0.05$, enhanced coupling was observed for the high-gamma amplitude of the IFG and the beta phase of amygdala (**c**, $n = 259$ channel pairs, $p = 0.039$). The comodulograms show differences in inter-regional phase-amplitude coupling between the painful and non-painful conditions. Warmer colors denote higher *t* values. Significant clusters were highlighted with black contours (corrected $p < 0.01$) or red contours (corrected $p < 0.05$) with insignificant frequency ranges presented with transparency. Source data are provided as a Source Data file.

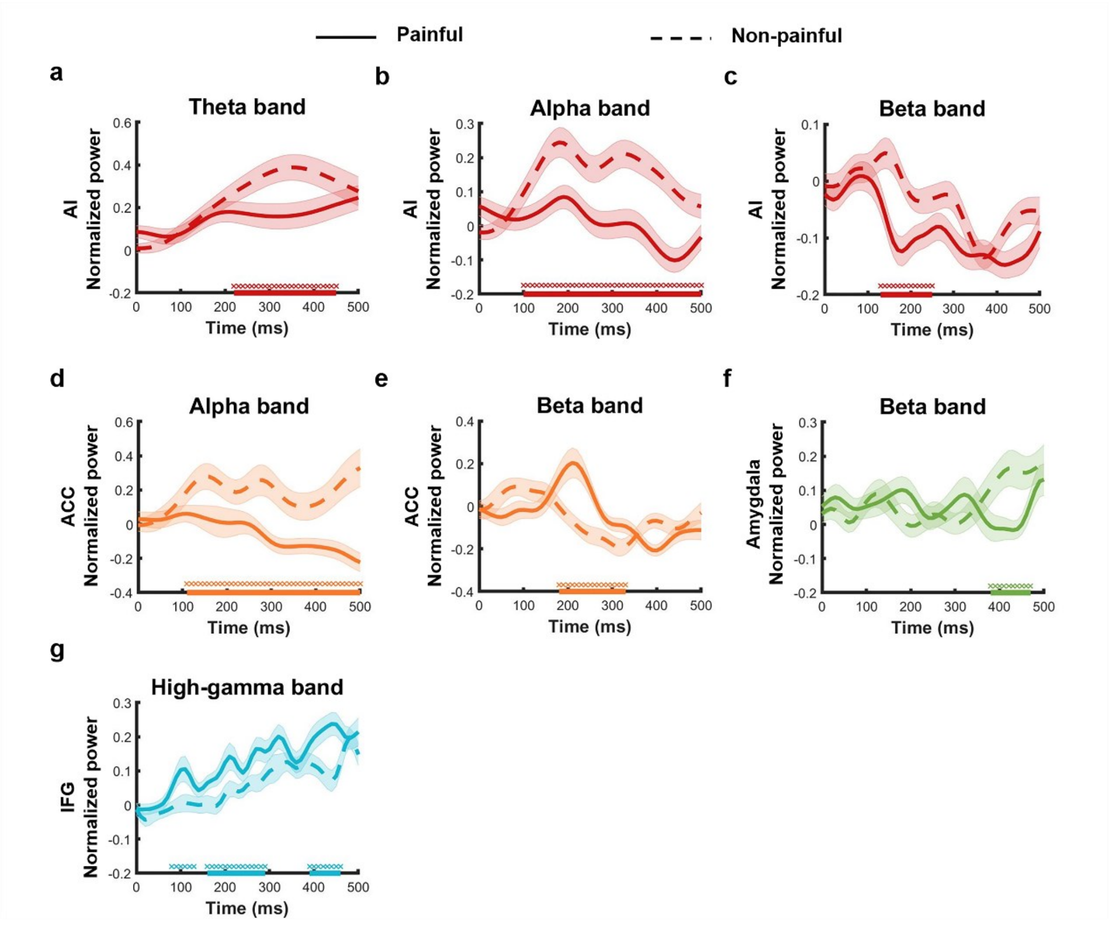

Supplementary Fig. 19 | ***Temporal profiles of AI, ACC, amygdala, and IFG neural activity, with the power time series smoothed by a sliding window of 50 ms.*** The theta/alpha/beta/high-gamma power averaged across all channels within each brain region was plotted as a function of time for the AI (n = 98 channels, **a**, theta band: p = 0.007; **b**, alpha band: p < 0.001; **c**, beta band: p = 0.008), ACC (n = 40 channels, **d**, alpha band: p < 0.001; **e**, beta band: p = 0.005), amygdala (n = 68 channels, **f**, beta band: p = 0.009) and IFG (n = 91 channels, **g**, high-gamma band, 80-130ms: p = 0.029; 160-290ms: p = 0.001; 390-460ms: p = 0.001). Time points with significant conditional power differences are highlighted with horizontal lines (corrected p < 0.01) or cross symbols (corrected p < 0.05) (one-tailed paired-t test for each timepoint, 1000 permutations, cluster-based permutation test, at least maintained for 50ms consecutively). Solid (painful condition) and dashed (non-painful condition) lines (shadows) indicate the mean (standard error) power across all channels for each time point. Source data are provided as a Source Data file.

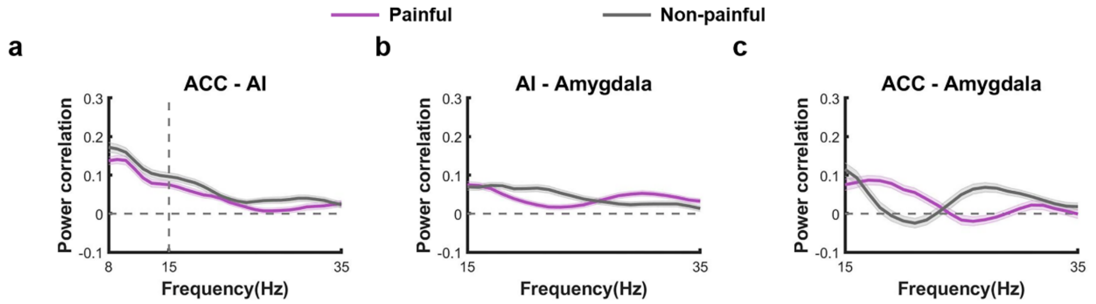

Supplementary Fig. 20 | ***Power correlations between ACC, AI and amygdala without Fisher-z-transformation.*** Purple (painful condition) and gray (non-painful condition) lines (shadows) indicate the mean (standard error) of frequency-resolved power correlations across all channel pairs for ACC-AI (**a**), AI-amygdala (**b**) and ACC-amygdala (**c**). Dashed vertical lines indicate boundaries between frequency bands. Source data are provided as a Source Data file.

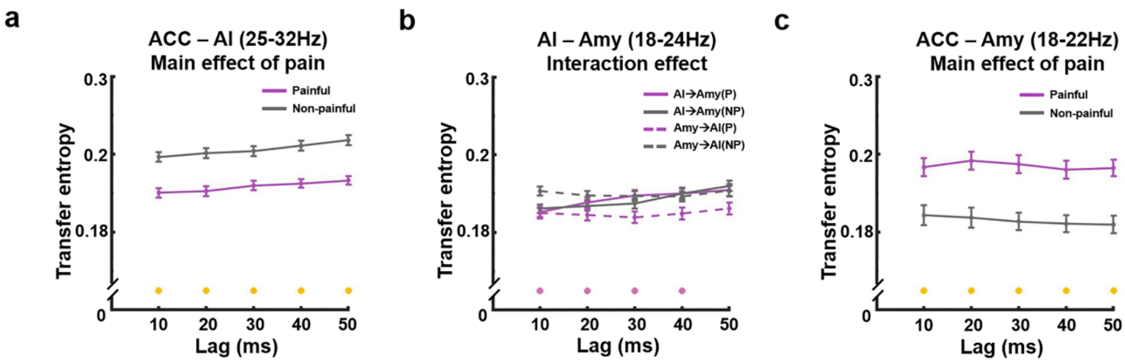

Supplementary Fig. 21 | ***Transfer entropy (TE) between ACC, AI, and amygdala with different lags.*** The main effect of pain for ACC-AI TE (**a**, $n = 234$ channel pairs, $p < 0.001$), the interaction effect for AI-amygdala TE (**b**, $n = 300$ channel pairs, $p = 0.004$), and the main effect of pain for ACC-amygdala TE (**c**, $n = 72$ channel pairs, $p = 0.002$) maintained when lag values ranging from 10 ms to 50 ms (1000 permutations, cluster-based permutation test, two-sided). Significant main effect of pain is highlighted with orange circles and significant interaction effect is highlighted with pink circles. Data are represented as mean ± SE. P = painful, NP = non-painful, Amy = amygdala. Source data are provided as a Source Data file.

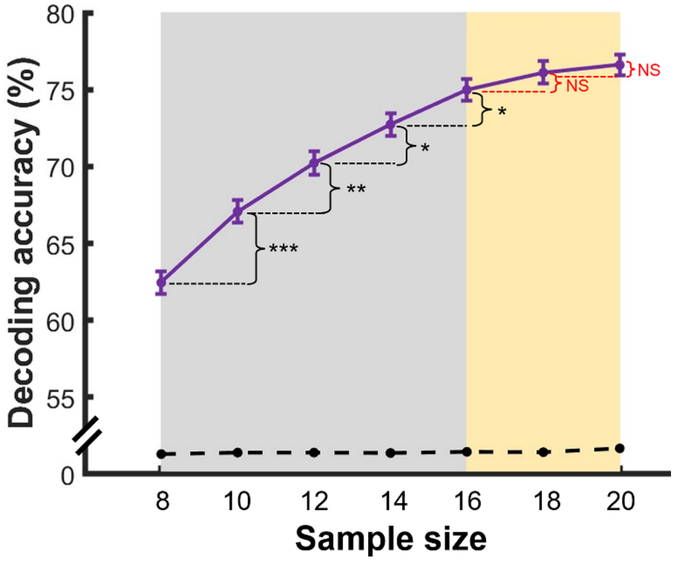

Supplementary Fig. 22 | ***Decoding accuracy with different sample size (number of trials).*** We decoded stimulus types with all neural features in different sample sizes. The purple solid-dotted line indicates the decoding accuracy (n = 200 sampling) across a range of 8 to 20 trials. Data are represented as mean ± SE. The black dash-dotted line indicates the statistical threshold of $p < 0.05$ (a less conservative threshold was used as this analysis was a further examination of our decoding results which had been reported in the main text, one-sided permutation test). A significant increase in decoding accuracy as the trial number increased: the decoding accuracy showed a significant improvement for every increment of two trials (highlighted by the grey shadow; two-sided independent sample t-test; 10 vs. 8: $t_{398} = 4.39$, $p = 1.48 \times 10^{-5}$; 12 vs.10: $t_{398} = 2.98$, $p = 0.003$; 14 vs. 12: $t_{398} = 2.34$, $p = 0.020$; 16 vs. 14: $t_{398} = 2.22$, $p = 0.027$) when the trial number was below 16. Critically, when the sample size researched 16 or above, further increases in samples did not lead to a significant improvement in decoding performance (highlighted by the yellow shadow; two-sided independent sample t-tests; 18 vs. 16: $t_{398} = 1.12$, $p = 0.262$; 20 vs. 18: $t_{398} = 0.18$, $p = 0.860$). *$p < 0.05$, **$p < 0.01$, ***$p < 0.001$, NS, insignificant. Source data are provided as a Source Data file.

Supplementary Table 1 | ***Statistical information of two-sided linear mixed-effect models testing for the intergroup differences in behavioral responses and subjective ratings***

**Behavioral indices**	***F* value (interaction)**	***p* value (interaction)**
Response time	0.05	0.823
Response accuracy	1.18	0.280
Rating of empathic strength	0.05	0.830
Rating of perceived pain intensity	0.70	0.406
Rating of unpleasantness	0.94	0.336

Supplementary Table 2 | *Statistical information of the cross-stimulus Pearson correlation (two-tailed) of subjective ratings between patients and healthy participants*

**Behavioral indices**	***r* value**	***p* value**
**Across all stimuli**		
Rating of empathic strength	0.97	9.73×10^{-13}
Rating of perceived pain intensity	0.97	1.02×10^{-12}
Rating of unpleasantness	0.96	3.11×10^{-11}
**Across all painful stimuli**		
Rating of empathic strength	0.71	0.022
Rating of perceived pain intensity	0.69	0.027
Rating of unpleasantness	0.79	0.007

Supplementary Table 3 | *Statistical information of the permutation tests about patient-normative similarity and healthy-normative similarity*

**Behavioral indices**	***p* value**
**Across all stimuli: Patient-normative similarity**	
Rating of empathic strength	$p < 0.001$
Rating of perceived pain intensity	$p < 0.001$
Rating of unpleasantness	$p < 0.001$
**Across all painful stimuli: Patient-normative similarity**	
Rating of empathic strength	$p < 0.001$
Rating of perceived pain intensity	$p < 0.001$
Rating of unpleasantness	0.003
**Across all stimuli: Healthy-normative similarity**	
Rating of empathic strength	$p < 0.001$
Rating of perceived pain intensity	$p < 0.001$
Rating of unpleasantness	$p < 0.001$
**Across all painful stimuli: Healthy-normative similarity**	
Rating of empathic strength	$p < 0.001$
Rating of perceived pain intensity	$p < 0.001$
Rating of unpleasantness	$p < 0.001$
**Across all stimuli: Patient-normative similarity minus Healthy-normative similarity**	
Rating of empathic strength	0.516
Rating of perceived pain intensity	0.124

**Behavioral indices**	***p* value**
Rating of unpleasantness	0.135
**Across all painful stimuli: Patient-normative similarity minus Healthy-normative similarity**	
Rating of empathic strength	0.287
Rating of perceived pain intensity	0.576
Rating of unpleasantness	0.583

Supplementary Table 4 | *Descriptive statistics of behavioral performances*

Behavioral indices		Non-painful condition	Painful condition
Response accuracy (%)	Mean ± SE	81.82 ± 3.58	87.73 ± 3.15
	Minimum	50	60
	Maximum	100	100
Response time (s)	Mean ± SE	1.02 ± 0.12	1.00 ± 0.10
	Minimum	0.28	0.33
	Maximum	2.99	1.69

Supplementary Table 5 | *Descriptive statistics of empathy-related ratings*

Dimension		Non-painful condition	Painful condition
Empathy-related subjective ratings of patients			
Empathy strength (ICC = 0.96)	Mean ± SE	7.10 ± 3.03	70.25 ± 5.19
	Minimum	0	20.64
	Maximum	46.20	100
Perceived pain intensity (ICC = 0.97)	Mean ± SE	5.18 ± 1.63	66.30 ± 5.32
	Minimum	0	17.10
	Maximum	20.1	96.9
Unpleasantness (ICC = 0.93)	Mean ± SE	8.61 ± 3.15	59.74 ± 7.02
	Minimum	0	1.80
	Maximum	42.35	97.50
Empathy-related subjective ratings of healthy participants			
Empathy strength (ICC = 0.98)	Mean ± SE	13.36 ± 2.60	75.00 ± 3.13
	Minimum	0	40
	Maximum	46.15	100
Perceived pain intensity (ICC = 0.98)	Mean ± SE	15.25 ± 3.44	82.19 ± 2.66
	Minimum	0	60.57
	Maximum	54.79	99.70
Unpleasantness (ICC = 0.96)	Mean ± SE	16.13 ± 3.06	76.47 ± 5.42
	Minimum	0.14	0
	Maximum	64.35	99.50

Note. ICC = intra-class coefficient.

Supplementary References

1. Hülsemann, M. J., Naumann, E. & Rasch, B. Quantification of phase-amplitude coupling in neuronal oscillations: comparison of phase-locking value, mean vector length, modulation index, and generalized-linear-modeling-cross-frequency-coupling. *Front. Neurosci.* **13,** 573 (2019).
2. Zhang, R. et al. Temporal-spatial characteristics of phase-amplitude coupling in electrocorticogram for human temporal lobe epilepsy. *Clin. Neurophysiol.* **128,** 1707-1718 (2017).
3. Mukamel, E. A. et al. A transition in brain state during propofol-induced unconsciousness. *J. Neurosci.* **34,** 839-845 (2014).
4. Chen, S. et al. Theta oscillations synchronize human medial prefrontal cortex and amygdala during fear learning. *Sci. Adv.* **7,** eabf4198 (2021).
5. Saez, I. et al. Encoding of Multiple Reward-Related Computations in Transient and Sustained High-Frequency Activity in Human OFC. *Curr. Biol.* **28,** 2889-2899 (2018).
6. Korzeczek, A., Neef, N. E., Steinmann, I., Paulus, W. & Sommer, M. Stuttering severity relates to frontotemporal low-beta synchronization during pre-speech preparation. *Clin. Neurophysiol.* **138,** 84-96 (2022).
7. Fernandez-Mendoza, J. et al. Insomnia is associated with cortical hyperarousal as early as adolescence. *Sleep* **39,** 1029-1036 (2016).
