# Supplementary material for: Intracranial EEG signals disentangle multi-areal neural dynamics of vicarious pain perception
Source: Nat Commun. 2024 Jun 18;15:5203. doi: 10.1038/s41467-024-49541-1 (PMC11189531; doi:10.1038/s41467-024-49541-1)
Supplement: Supplementary file 3 — Description of Additional Supplementary Files [file 41467_2024_49541_MOESM3_ESM.pdf]

Description of Additional Supplementary Files

File name: Supplementary Data 1
Description: Descriptive statistics of trial number across channels or channel pairs. For each analysis, we described the mean, standard error, minimum and maximum of trial number across channels or channel pairs.

File name: Supplementary Data 2
Description: Channel coordinate within the ACC, AI, amygdala and IFG for all patients in the MNI space. For each channel, we depicted its corresponding patient and MNI coordinates and also the MNI coordinate of its corresponding nearest white-matter neighbor reference channel.

File name: Supplementary Data 3
Description: Summary of channel/channel-pair information. We summarized how many channels or channel pairs each patient had for each of brain region of interest or region pair of interest. At the last row, we described the total number of channels or channel pairs across all patients for each of brain region of interest or region pair of interest.
