# Supplementary material for: Intracranial EEG signals disentangle multi-areal neural dynamics of vicarious pain perception
Source: Nat Commun. 2024 Jun 18;15:5203. doi: 10.1038/s41467-024-49541-1 (PMC11189531; doi:10.1038/s41467-024-49541-1)
Supplement: Supplementary file 7 — Reporting Summary [file 41467_2024_49541_MOESM7_ESM.pdf]

nature portfolio

Corresponding author(s): Yina MA

Last updated by author(s): May 17, 2024

Reporting Summary

Nature Portfolio wishes to improve the reproducibility of the work that we publish. This form provides structure for consistency and transparency in reporting. For further information on Nature Portfolio policies, see our Editorial Policies and the Editorial Policy Checklist.

Statistics

For all statistical analyses, confirm that the following items are present in the figure legend, table legend, main text, or Methods section.

n/a	Confirmed	
☐	☒	The exact sample size (*n*) for each experimental group/condition, given as a discrete number and unit of measurement
☐	☒	A statement on whether measurements were taken from distinct samples or whether the same sample was measured repeatedly
☐	☒	The statistical test(s) used AND whether they are one- or two-sided<br>*Only common tests should be described solely by name; describe more complex techniques in the Methods section.*
☐	☒	A description of all covariates tested
☐	☒	A description of any assumptions or corrections, such as tests of normality and adjustment for multiple comparisons
☐	☒	A full description of the statistical parameters including central tendency (e.g. means) or other basic estimates (e.g. regression coefficient) AND variation (e.g. standard deviation) or associated estimates of uncertainty (e.g. confidence intervals)
☐	☒	For null hypothesis testing, the test statistic (e.g. *F*, *t*, *r*) with confidence intervals, effect sizes, degrees of freedom and *P* value noted<br>*Give P values as exact values whenever suitable.*
☒	☐	For Bayesian analysis, information on the choice of priors and Markov chain Monte Carlo settings
☐	☒	For hierarchical and complex designs, identification of the appropriate level for tests and full reporting of outcomes
☐	☒	Estimates of effect sizes (e.g. Cohen's *d*, Pearson's *r*), indicating how they were calculated

Our web collection on statistics for biologists contains articles on many of the points above.

Software and code

Policy information about availability of computer code

Data collection	iEEG data were collected with the Nicolet electroencephalogram system (256 channel amplifier, the Chinese PLA General Hospital), Nihon Koheden system (256 channel amplifier, Beijing Tiantan Hospital, Capital Medical University) and Micromed system (128 channel amplifier, Xuanwu Hospital, Capital Medical University). Behavioral data were collected with Psychtoolbox (version 3.0.14) and MATLAB R2018a.
Data analysis	Behavioral data were analyzed with MATLAB R2020b, R (version 4.1.3), SPSS (version 20),bruceR package (version 0.8.6) and irr package (version 0.84.1). iEEG data were analyzed with MATLAB R2020b, Fieldtrip toolbox (version 20210709), R (version 4.1.3), Neuroscience Information Theory toolbox (available at https://github.com/nmtimme/Neuroscience-Information-Theory-Toolbox) and Statistics and Machine Learning Toolbox (version 12.0). Channel locations were visualized though BrainNet Viewer (version 20181219).

For manuscripts utilizing custom algorithms or software that are central to the research but not yet described in published literature, software must be made available to editors and reviewers. We strongly encourage code deposition in a community repository (e.g. GitHub). See the Nature Portfolio guidelines for submitting code & software for further information.

Data

Policy information about availability of data

All manuscripts must include a data availability statement. This statement should provide the following information, where applicable:

- Accession codes, unique identifiers, or web links for publicly available datasets
- A description of any restrictions on data availability
- For clinical datasets or third party data, please ensure that the statement adheres to our policy

The raw and preprocessed iEEG data generated in this study have been deposited in a local database. These data are available under restricted access as they contain personally identifiable information and patients have not consented to data distribution. Access can be obtained from the corresponding author upon reasonable request. Source data are provided with this paper.

Research involving human participants, their data, or biological material

Policy information about studies with human participants or human data. See also policy information about sex, gender (identity/presentation), and sexual orientation and race, ethnicity and racism.

Reporting on sex and gender	We recruited both male and female participants. Our findings could apply to both males and females. Our study design did not include the variable sex as a variable of interest. We did not perform a sex-based analysis due to the limited number of participants.
Reporting on race, ethnicity, or other socially relevant groupings	Our study design did not include the race, ethnicity or other social variables as variables of interest.
Population characteristics	For the iEEG study, 29 participants undergoing iEEG for the purpose of tracking drug-resistant epilepsy were invited to participate in this study on a volunteer basis. All participants recruited in the current study had no history of psychiatric disorders, head trauma, or encephalitis. Patients did not take pain medication several hours prior to the iEEG recording of the pain judgment task and were not experiencing any physical pain during the iEEG recording. The patient selection was based on two inclusion criteria: i) having electrodes in the ACC, AI, amygdala, or IFG contralateral to or outside of the epileptogenic zone; and ii) achieving a response accuracy above 50% in the pain judgment task. Based on these criteria, one patient was excluded due to a low response accuracy (45%) in the pain judgment task, and six patients were excluded because no electrodes were implanted in the regions of interest. The remaining 22 patients were included in the behavioral and neural analysis of the pain judgment task (13 males, age = 25.73 ± 2.07 years old).Additionally, we recruited a healthy participant sample whose gender and age distributions were comparable to those of the patient sample (n = 22; 9 males, age = 23.18 ± 2.38 years old) .
Recruitment	The epilepsy patients were recruited during their hospital stay for the continuous monitoring of seizures. The independent sample of 22 healthy participants was recruited in this study as paid volunteers through the on-campus flyer recruitment. No self-selection bias was involved in the participant recruitment.
Ethics oversight	Electrode localizations were exclusively determined by clinical needs. We prioritized and maintained the integrity of clinical care during conducting the current study. All patients provided informed consent after the experimental procedure had been fully explained, and were acknowledged their right to withdraw at any time during the study. The experimental design and procedures adhered to the standards set by the Declaration of Helsinki and were approved by the local Institutional Review Board of each hospital where the patients were tested (i.e., the Chinese PLA General Hospital: S2021-394-02, Beijing Xuanwu Hospital: ClinRes No.2022018, and Beijing Tiantan Hospital: KY 2020-080-02).

Note that full information on the approval of the study protocol must also be provided in the manuscript.

Field-specific reporting

Please select the one below that is the best fit for your research. If you are not sure, read the appropriate sections before making your selection.

☒ Life sciences ☐ Behavioural & social sciences ☐ Ecological, evolutionary & environmental sciences

For a reference copy of the document with all sections, see nature.com/documents/nr-reporting-summary-flat.pdf

Life sciences study design

All studies must disclose on these points even when the disclosure is negative.

Sample size	22 patients were included in the behavioral and neural analysis of the pain judgment task (13 males, age = 25.73 ± 2.07 years old). Data from neurosurgical patients participating in research were collected over 4 years. No sample size calculation was performed, but our sample sizes are similar to those reported in previous iEEG publications. The main analyses were conducted in regions of interest at the channel level (40 to 98 channels per region) or region pairs of interest at the channel-pair level (72 to 459 channel pairs), which are similar to (or larger than) that of many previous iEEG studies. A post-hoc power analysis (two-sided, paired-t tests, alpha error = 5%) confirmed that we had sufficient power

(86.94%) to detect medium effect sizes (d = 0.5) even with the minimum number of channels (n = 40).

Data exclusions

The patient selection was based on two inclusion criteria: i) having electrodes in the ACC, AI, amygdala, or IFG contralateral to or outside of the epileptogenic zone; and ii) achieving a response accuracy above 50% in the pain judgment task. Based on these criteria, one patient was excluded due to a low response accuracy (45%) in the pain judgment task, and six patients were excluded because no electrodes were implanted in the regions of interest. The remaining 22 patients were included in the behavioral and neural analysis of the pain judgment task (13 males, age = 25.73 ± 2.07 years old).

We invited all patients to a post-iEEG session to measure the empathic strength and other empathy-related subjective ratings to perceived pain in others after the iEEG recording. No data were excluded, but the subjective ratings of six patients were missing as the six patients were unwilling to or failed to complete the post-iEEG session. Thus, the behavioral and neural analysis of subjective ratings were conducted on the remaining 16 patients (10 males, age = 24.63 ± 2.35 years old).

For the analysis of iEEG data, all channels underwent a quality check and were discarded if any of the following criteria were met: 1) variances were five times greater than the median variance across all channels within the same category (gray matter channels or white matter channels); and 2) the number of jumps between consecutive data points larger than 100 µV was more than three times the median number of such jumps across all channels within the same category. All the remaining channels were also visually inspected to ensure that all bad channels had been removed.

Epileptic charges were identified via an automatic assessment: 1) the envelope of the unfiltered signal was five standard deviations away from the baseline (i.e., the whole time series); or 2) the envelope of the filtered signal (band-pass filtered between 25-80 Hz) was six standard deviations away from the baseline. Our neural analysis focused on the presentation phase of the painful or non-painful stimuli and therefore each trial was epoched from 200 ms before to 500 ms after the stimulus onset. Any epoch containing epileptic charges was removed from further analyses and any channel with more than 30% epochs removed from either painful or non-painful conditions was excluded. Finally, we visually screened all channels for epileptic charges and removed those with too many remaining artifacts. All visual inspections were performed while blinded to the experimental conditions.

Replication

The iEEG data are a rare dataset. Due to the inherent difficulty and invasiveness of iEEG acquisition, we could not provide a direct replication of the results using an independent sample. Yet, the main observed effects in the spectro-temporal power, power correlations, and PAC analyses were replicated when we changed statistical models (using linear mixed-effect model) and referencing schemes (re-reference to the closest white matter channels).

Randomization

Randomization was not applicable to the study since there was no group of subject to randomize. However, in the iEEG experiment, experimental conditions were randomized, as detailed in the methods section.

Blinding

Blinding was not applicable to the study since there was no group allocation in our study.

Reporting for specific materials, systems and methods

We require information from authors about some types of materials, experimental systems and methods used in many studies. Here, indicate whether each material, system or method listed is relevant to your study. If you are not sure if a list item applies to your research, read the appropriate section before selecting a response.

Materials & experimental systems

n/a	Involved in the study	
☒	☐	Antibodies
☒	☐	Eukaryotic cell lines
☒	☐	Palaeontology and archaeology
☒	☐	Animals and other organisms
☒	☐	Clinical data
☒	☐	Dual use research of concern
☒	☐	Plants

Methods

n/a	Involved in the study	
☒	☐	ChIP-seq
☒	☐	Flow cytometry
☒	☐	MRI-based neuroimaging
